# Editorial: Leeway to Operate With Plant Genetic Resources

**DOI:** 10.3389/fpls.2020.00911

**Published:** 2020-07-24

**Authors:** Dennis Eriksson, Rodomiro Ortiz, Richard G. F. Visser, Juan Antonio Vives-Vallés, Humberto Prieto

**Affiliations:** ^1^Department of Plant Breeding, Swedish University of Agricultural Sciences, Uppsala, Sweden; ^2^Plant Breeding, Wageningen University & Research, Wageningen, Netherlands; ^3^Private Law Department, University of the Balearic Islands, Palma de Mallorca, Spain; ^4^Agro-Food Production and Protection Department, Agro-Environmental and Water Economics Institute, Palma de Mallorca, Spain; ^5^Institute of Agricultural Research, Santiago, Chile

**Keywords:** access and benefit-sharing, biosafety, intellectual property, plant genetic resources, research and breeding

Different legal frameworks are applicable to the use of genetic resources (GR). These can broadly be categorized into (1) access and benefit-sharing (ABS), (2) biosafety aspects related to the technologies for improving the genetic material, and (3) intellectual property (IP) systems including plant variety rights (PVR) and patents specific to the plant innovation sector. With scientific and technical progress in research and breeding, as well as expanding internationalization, legal frameworks have become increasingly complex in the past few decades. In this context, the Research Topic “Leeway to operate with plant genetic resources” addresses the latest and most pertinent legal issues related to the use of GR in plant research and breeding. The contributions are summarized here and put into the larger societal and legal context that modern-day plant geneticists are facing.

## Access and Benefit-Sharing

ABS is a framework that aims to distribute fairly the benefits arising from the utilization of GR between users and providers. The basic principles are drawn in the Convention on Biological Diversity (CBD) and its supplementary protocol, the Nagoya Protocol (https://cbd.int/abs/). The access to GR also considers the related traditional knowledge and is based on prior informed consent and mutually agreed terms.

There is a wide disparity in how the Nagoya Protocol is implemented in different countries, which is challenging for users. Sirakaya et al. reviews the ABS framework across 20 provider countries, identifying common regulatory elements and follows up with stakeholder interviews. These show that opinions on the benefits of various ABS regulatory mechanisms differ between provider countries and industrial users, though there are some common grounds. One significant detail is that most users oppose the inclusion of digital sequence information (DSI) within the subject matter, contrary to most provider countries. We note that FAO acknowledges that DSI increases the understanding of molecular biology and evolution as well as taxonomy and identifying species, thus facilitating GR conservation and use. Aubry et al. elaborates further on the ongoing debate about the sharing and mining of freely accessible sequencing data. In his view, DSI of plant genetic resources for food and agriculture (PGRFA) should be under an “*efficient, resilient, decentralized*” and reasonable governance model that ensures its fair use.

Brink and van Hintum address the perspective of collection holders, showing the challenges faced by gene banks for acquiring and sharing GR while complying with the various international and national regulations. They argue that gene banks must set up appropriate protocols for documenting every accession's origin and the condition for its use and further distribution, while countering complexity to avoid a decrease in access to PGRFA. Overall, it is important to ensure fair and equitable ABS negotiations between providers and users. Deplazes-Zemp et al. brings an ethical perspective, arguing that there are five types of justice related to this subject: distributive, commentative, recognitive, reparative, and procedural. According to the author, it is important that both users and providers are aware of these justice types and the way the use of GR poses particular challenges.

## Biosafety

The products of gene technologies, such as genetically modified organisms (GMOs) are subject to a specific biosafety legislation, in most jurisdictions. building on principles established by the Cartagena Protocol to the CBD (https://bch.cbd.int/protocol/). The legal status of the products of new breeding techniques (NBTs) has been subject to many discussions, as the resulting products may or may not be encompassed by the GMO definition, depending on the jurisdiction.

A landmark judgment from the Court of Justice of the European Union (CJEU) in July 2018 (case C-528/16) means that the products of site-directed mutagenesis will be subject to the same legal provisions as genetically modified organisms (GMOs). There are however discussions on the applicability of the CJEU judgment to the variety of NBT products. Vives-Vallés and Collonnier provide a legal interpretation of the judgment, relating it to relevant scientific papers published in the aftermath. Their article concludes that certain products of NBTs may be exempted from the scope of Directive 2001/18/EC, despite the CJEU judgment being commonly interpreted otherwise, and sketches a limited legislative proposal to achieve certainty and suggesting which NBT products could be exempted and under what circumstances. Holme et al. argues that the CJEU assumption that targeted mutagenesis “*makes it possible to produce GM varieties at a rate and in quantities unlike those resulting from random mutagenesis*,” is incorrect. Technical developments including TILLING has led to a convergence between the two types of mutagenesis in terms of output, with the main differences being the precision of mutation site and the number of off-target mutations.

Turning to the economics of regulating NBT products, Wesseler et al. compares theoretical advantages and disadvantages with different regulatory approaches. A survey among Dutch plant breeding companies show that these are optimistic the prospect that a more relaxed legislation will be implemented in the EU, despite having experienced a negative impact on competitiveness and on investments due to the CJEU judgment on mutagenesis. Jin et al. present an example of costs in delaying technology adoption. By assessing the impact caused by postponed authorization for the use of *Bt* rice in China, the authors model the costs by filling an information gap regarding foregone benefits of lower pesticide use and the spill-over effect by delaying technology adoption. They conclude that delaying *Bt* rice introduction has come at a substantial economic cost (both direct as well as in terms of human health and environmental costs).

The Green Revolution based on crop genetics along with advanced agronomy led to miraculous harvests in Asia and Latin America, but largely bypassed sub-Saharan Africa. The ongoing gene revolution should therefore bring benefits to this region. Komen et al. show how the authorization of transgenic crops release is managed, drawing examples from five African countries. They highlight challenges and lessons learnt and propose policy recommendations facilitating the adoption of emerging biotechnology for plant breeding in Africa. It has however been recognized elsewhere in the literature that the global influence of EU policies should be considered. The overall process for risk assessment and risk management of GMOs in the EU has been criticized as being unnecessary politicized and, though the part with the science-based system for risk assessment is overall sound, certain improvements are envisaged by Chatzopoulou et al. The authors compare the procedure in the European Food Safety Authority (EFSA) with that of the European Medicines Agency (EMA), and suggest that a more balanced geographical distribution of experts in the EFSA GMO panel may minimize overall politicization of decision-making.

## Intellectual Property

Current technical developments are posing challenges to the IP/PVR systems in plant breeding. Definitions need to be re-established and the impact of the evolution of systems for patents and for plant breeders' rights need to be analyzed in terms of market structure and competition. One example is the concept of Essentially Derived Variety (EDV), for which UPOV is currently revisiting their explanatory note. Krieger et al. explore the concept and assess whether the use of NBTs invariably leads to an EDV. The authors deliver a legal interpretation of two related notions, namely, the “breeder's exemption” and EDV, considering the wording of the provisions, the historical background and the evolution, including also assertions from case law from several UPOV members. Several elements are discussed, concluding that the EDV concept should encompass cultivars obtained through the application of NBT only, when no further significant breeding steps have been taken.

In terms of market structure and innovation, Wozniak et al. analyse the current situation and prospects of rapeseed in the EU taking Poland and Germany as benchmarks. The study considers several IP as well as agronomic factors and analyses their evolution overtime, describing patterns regarding the opportunities of rapeseed. Though an analysis of IP shows an innovation potential, the authors are concerned that the CJEU judgment on mutagenesis may have a negative impact on the expansion of rapeseed cultivation.

Following concerns about the increasing impact of patenting and of concentration in the seed sector, an Open Source Seed (OSS) model has been proposed in recent years. Louwaars et al. investigates this model and its impact on the breeder's exemption specifically and on the open innovation character of the PVR system in general. Focusing on two examples from Germany and USA, the author suggests that OSS models add additional pressure on the breeder's exemption, which may already be restrained by patents and biodiversity schemes, thus concluding that the breeder's exemption is an appropriate solution to ensure the access to genetic material.

Altogether, these articles illustrate the complexity of legal frameworks that plant researchers and breeders need to be aware of and comply with. Scientific and technological progress is enhancing our capacity to work with GR and causes restructuring of markets and competition and re-definitions of established concepts. We hope this Research Topic will provide a valuable resource for all stakeholders, including scientists, legal researchers, and practitioners that wish to stay up to date in this field.

## Author Contributions

All authors listed have made a substantial, direct and intellectual contribution to the work, and approved it for publication.

## Conflict of Interest

The authors declare that the research was conducted in the absence of any commercial or financial relationships that could be construed as a potential conflict of interest.

